# Hepatic Abscess Following Acute Appendicitis

**DOI:** 10.7759/cureus.26867

**Published:** 2022-07-14

**Authors:** Tavish E Ward, Rohan K Mangal, Thor S Stead, Latha Ganti

**Affiliations:** 1 Biology, Trinity Preparatory School, Winter Park, USA; 2 Medicine, University of Miami Miller School of Medicine, Miami, USA; 3 Medicine, Warren Alpert Medical School of Brown University, Providence, USA; 4 Emergency Medicine, University of Central Florida College of Medicine, Orlando, USA; 5 Emergency Medicine, Envision Physician Services, Plantation, USA; 6 Emergency Medicine, HCA Florida Ocala Hospital, Ocala, USA

**Keywords:** liver abscess drainage, abdominal pain in females, appendicitis, hepatic abscess, pyogenic hepatic abscess

## Abstract

The authors present the case of a 29-year-old female diagnosed with acute appendicitis who underwent an uneventful laparoscopic appendectomy. Three weeks later, she returned to the emergency department with fevers, abdominal pain, chills, and nausea. Laboratory analysis revealed elevated liver enzymes and leukocytosis, and a computed tomography scan of the abdomen revealed a liver abscess. Interventional radiology placed an 8 French drainage catheter in the hepatic abscess and drained 40cc of purulent fluid. A culture of the abscess fluid revealed Streptococcus constellatus, Bacteroides fragilis, and Bacteroides ovatus. We believe appendicitis causes hematogenous spreading of bowel organisms along the portal vein, which is seeded to the liver.

## Introduction

A hepatic abscess is a pus-filled mass inside the liver, with the three main classifications being pyogenic, amoebic, and fungal. Liver abscesses are extremely rare and have an incidence rate of about 0.0023% [1]. Men and people over 65 are more likely to get pyogenic liver abscesses and anyone with a history of malignancy, alcoholism, diabetes, and liver transplants [1]. Pyogenic liver abscesses are often caused by some bacteria, the most common ones being E. Coli and Streptococcus milleri [2]. There are also a considerable number of cases that are culture negative, although treatment and aftermath are mostly the same. In both culture-negative and culture-positive liver abscesses, symptoms usually include fevers, right upper quadrant pain, nausea, and weight loss [3]. The optimal treatment is drainage of the abscess [4], with advances in technology reducing the mortality rate to around 6% [5]. The authors present a case of a 29-year-old female who was diagnosed with a hepatic abscess three weeks after an appendectomy.

## Case presentation

A 29-year-old female presented to the emergency department (ED) complaining of profuse vomiting and abdominal pain for about a day. She claimed to have had about 30 episodes of vomiting before coming to the ED. She had no past medical history and was not on any daily medications. The only medication she took to help alleviate symptoms was naproxen. Her vital signs were unremarkable. Lab results revealed leukocytosis with a left shift and elevated neutrophils, and a computed tomography (CT) scan of the abdomen was consistent with acute appendicitis. She underwent an uneventful laparoscopic appendectomy and was discharged on day three.

Five days later, the patient returned to the ED due to abdominal pain and bleeding at the incision site. It was determined that she presented with normal postoperative pain and minimal bleeding from the incision site and was discharged with outpatient surgical follow-up. A CT scan was not obtained during this visit.

**Figure 1:**
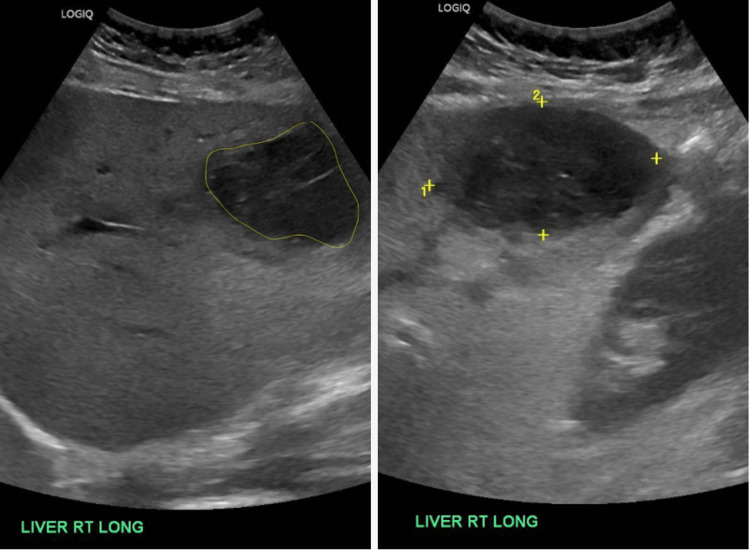
Sonogram demonstrating heterogeneous right hepatic mass (circles on left, and hatchmarks on right) and an additional smaller hypoechoic lesion, suspicious for underlying malignancy.

**Figure 2:**
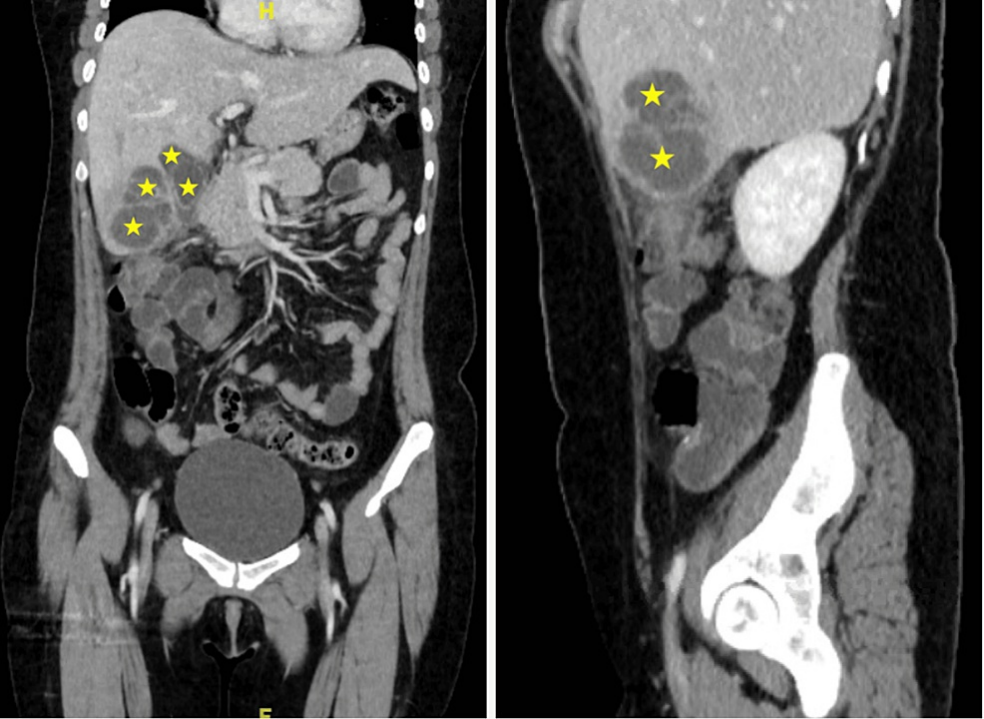
Coronal (left) and saggital (right) views of Computed tomography scan demonstrating liver abscesses (stars)

Three weeks after the patient underwent appendectomy, she returned to the ED complaining of abdominal pain, nausea, chills, and fever for about four days. She described the pain as severe and rated it a 10/10. She described a sharp stabbing quality that was non-radiating and worse with movement, eating, and breathing. The patient stated she had been taking acetaminophen with good effect before the symptoms began but were not very effective afterward. Laboratory results revealed elevated liver enzymes and leukocytosis [Table 1]. The initial impression of the ultrasound was actually a malignancy [Figure 1], but a CT scan of the abdomen revealed interval development of heterogeneous multi-septated lesion within the right hepatic lobe consistent with an abscess given the inflammation in the adjacent gallbladder region [Figure 2].

**Table 1:** Significant Lab Results (before and after)

	Before (at the time of appendicitis)	After (at the time of liver abscess)	Reference Value
Alanine transaminase (ALT)	34	52	15-37 unit/L
Aspartate transaminase (*AST*)	49	110	12-78 unit/L
Total Alkaline Phosphatase	144	405	46-117 unit/L
White Blood Cell count	14.7	16.7	4.0-10.5 10^3/u
Neutrophils (%)	96.5	80.7	34.0-71.1%

Interventional radiology placed an 8 French drainage catheter in the hepatic abscess and drained 40cc of purulent fluid. She was given two grams of ceftriaxone and 500 mg of metronidazole intravenously, as well as morphine and ondansetron for pain and vomiting. A culture of the abscess fluid revealed Streptococcus constellatus, Bacteroides fragilis, and Bacteroides ovatus. The patient worsened before she improved, with continued fevers and leukocytosis as high as 26.2K/u. On day seven, she was afebrile, had normal white blood cell counts, and had less than 5cc drainage from the abscess, leading to her discharge.

## Discussion

Hepatic abscesses are extremely rare, affecting only about 0.0023% of the population [1]. Even more rare is a pyogenic liver abscess resulting from appendicitis. Varying results have been found when attempting to determine how prevalent appendicitis is in causing a pyogenic liver abscess. While one study has found that appendicitis only causes about 1% of pyogenic liver abscesses [1], another study found that it causes almost 10% [6]. The patient presented, being a 29-year-old female, is an oddity compared to the average spectrum of patients diagnosed with pyogenic liver abscess, with the risk factors for this disease including the male gender and older age [1,7].

The patient’s appendicitis is believed to have caused the hematogenous spreading of bowel organisms along the portal vein, which is seeded in the liver. A culture of the abscess fluid revealed Streptococcus constellatus, Bacteroides fragilis, and Bacteroides ovatus. All these bacteria are found in the Gastrointestinal Tract, and Streptococcus constellatus is commonly associated with appendicitis [8]. These findings are further evidence of seeding to the liver due to appendicitis, resulting in the abscess.

A rare aspect of this case is the time when the liver abscess was found. In other similar cases, the liver abscess was usually found before or during the appendicitis diagnosis [2,6,9,10]. In this case, however, the abscess was found a few weeks after the completed appendectomy.

Interestingly, the initial diagnosis through ultrasonography was a malignancy, and it was only after the CT scan of the abdomen that the abscess was revealed. Studies have shown CT scans to be slightly more accurate when diagnosing pyogenic liver abscess [1], and the presented case demonstrates these findings.

When dealing with pyogenic liver abscess, drainage and antibiotics are essential for treatment. Drainage is often done under the guidance of a CT or US. If the abscess is under five centimeters, then needle aspiration is usually all that’s warranted. However, for abscesses over five centimeters, a catheter placement may be more efficacious [1]. Catheter drainage was used in the presented case. If drainage fails, surgery may be necessary. Antibiotics should also be administered to a patient presenting with a liver abscess. In this case, ceftriaxone and metronidazole were given. The patient experienced significant clinical improvement following the drainage and antibiotics and was discharged in seven days.

## Conclusions

In the presented case, a 29-year-old female was diagnosed with a hepatic abscess three weeks after undergoing an appendectomy. It is suspected that appendicitis causes hematogenous spreading of bowel organisms along the portal vein, which is seeded in the liver. Although this occurrence is rare, it is essential to recognize a hepatic abscess as a potential complication of appendicitis.
